# 2646. Characterization of Respiratory Infections Before and After the Emergence of SARS-CoV-2: Beyond the “Tripledemic”

**DOI:** 10.1093/ofid/ofad500.2258

**Published:** 2023-11-27

**Authors:** Michaela Powell, Jay Jones

**Affiliations:** bioMérieux Inc., Salt Lake City, Utah; bioMérieux Inc., Salt Lake City, Utah

## Abstract

**Background:**

Respiratory infections place a large burden on society. The CDC reported “at least 26 million illnesses, 290,000 hospitalizations, and 19,000 deaths from flu” for the 2022-2023 season. While these estimates give evidence of this burden, other pathogen contributors are not represented.

The BioFire® system is a multiplex PCR diagnostic system from bioMérieux. The BioFire respiratory panels test for up to 22 pathogens organized into 10 target groups. BioFire® Syndromic Trends (Trend) is a network of deidentified patient test data from sites that elect to contribute. Using Trend data, we characterize respiratory infections for more pathogens than the CDC’s flu reporting and evaluate the impact of the emergence of SARS-CoV-2 on the pathogen distribution during respiratory season.

**Methods:**

All patient runs in Trend from US sites between 1/4/2015 and 4/15/2023 were considered; > 1,500,000 runs. Respiratory seasons were defined using data available from the CDC’s FLUVIEW. The season’s start date was defined as the week when weighted ILI surpassed the National Baseline, and the end date was the week when it fell below the baseline. A 2020-2021 season is not included, because it did not surpass the baseline.

Filtering Trend data to respiratory seasons, we considered positivity of 9 target groups aggregated by pre-pandemic and post-pandemic seasons - excluding SARS-CoV-2 from post-pandemic data. Fisher’s Exact Test was used to test whether the distribution of the target groups has changed since the introduction of SARS-CoV-2. We then compared the pre-pandemic and post-pandemic positivity of each target group.

BioFire® Syndromic Trends Respiratory Weekly Positivity

**Figure:**
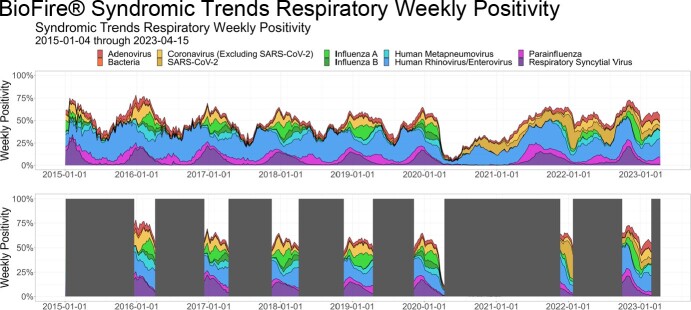

Observed weekly positivity of BioFire® Syndromic Trends respiratory patient runs from January 4, 2015 through April 15, 2023 by target group. Top panel displays all data. Bottom panel displays data for respiratory seasons.

**Results:**

The distribution of target groups during respiratory season has changed since the introduction of SARS-CoV-2 (p-value < 0.001). When considering the pre-pandemic and post-pandemic positivity of each target group, three have increased since the introduction of SARS-CoV-2 while six have decreased. The observed total increase was 6.49%, and the observed total decrease was 10.38%.

BioFire® Syndromic Trends Pre-Pandemic vs. Post-Pandemic Respiratory Season Positivity

**Figure:**
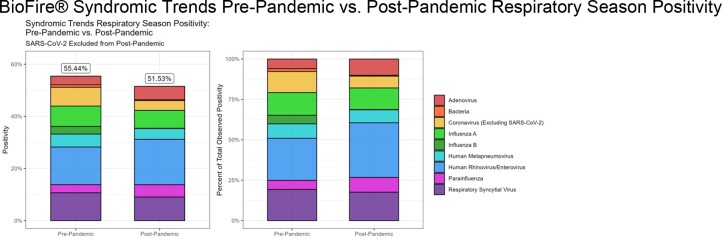

BioFire® Syndromic Trends respiratory data for pre-pandemic and post-pandemic aggregated respiratory seasons. Left panel displays the observed positivity of each target group. Right panel displays the percent of total observed positivity that each target group comprises.

BioFire® Syndromic Trends Pre-Pandemic vs. Post-Pandemic Target Group Positivity

**Figure:**
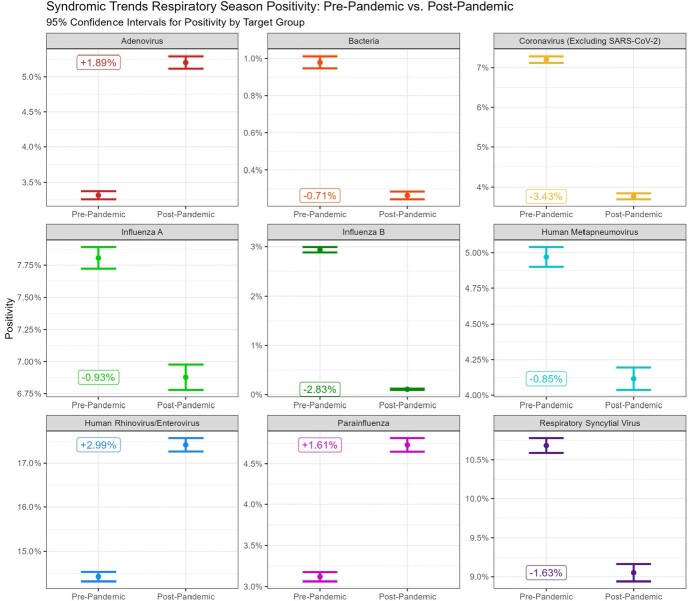

BioFire® Syndromic Trends target group positivity for Pre-Pandemic and Post-Pandemic aggregated respiratory season data with 95% confidence intervals.

**Conclusion:**

The BioFire® Syndromic Trend system allows for visibility into the role a larger set of pathogens play in respiratory season than what is available via the CDC’s ILI monitoring. Using this, we see that SARS-CoV-2 has changed the distribution of other respiratory pathogens.

**Disclosures:**

**All Authors**: No reported disclosures

